# Supplementary material for: Chromothripsis during telomere crisis is independent of NHEJ, and consistent with a replicative origin
Source: Genome Res. 2019 May;29(5):737–49. doi: 10.1101/gr.240705.118 (PMC6499312; doi:10.1101/gr.240705.118)
Supplement: Supplemental Material [file supp_gr.240705.118_Supplemental_file_1.zip › contigs/annotated_contigs/DB56/contig.2.DB56_length_750_mean_cov_8.32266666667.docx]

**DB56_length_750_mean_cov_8.32266666667**

```
AATGTTTTTGTTTTCTCAGACAACTACTGATTTTGCATTTATAAGTGTCAAGTGCCATGACTTTTTGCACAGATATACCCATACATGCA
 >chr4:163086867-163087194 + E=7e-182 p=2e-02
TGTATTATGAGTTCTTCAATTCCCAAAGTCATTTTAATAAAGGTGGCAGACACCATGATATGTCAGGAAAGATGATCTGTGGGGGCAGA

TGGGTGTTGCACAATTACAATTACAATGGAGCCAACTGTATAACTTGAACTTAGGCTGGCTACGGCGCACATGATATGCTTCTTTTGGG

AACATCGCATAGCACACTTTGGTCTGCTTCATGCCAATGTGCTGAAATGTCAGAGAAAT|C|ACATATGTTTTATTTCATTGTAATGTT
                                                            >chr4:163087962-163088386 +
CTATTTAAAATGTCTATTAGGTATATGTCTACTGTGGTAAAGGGCATTTAAATACAGTATTTTGTTATTGAAAGAAATATTGACACAAT
 E=3e-240
ATATTTATATTAAATTTCTTAGCTAATTCCAAATTGTGACCTTAAACAAATTTATATGTGTGCATGTGTGTGTGTGTGTGTGTGTGTGT

TTATGTGGGTAGGATATACTAAAGAGAATTTTCAAATATGTTTTCTTAAAATTATGGAAATAAAATTTAAGATATATGAATTAAAAACT

CACTACTAATAAATAAACCCGCAAATTAAAGAACACTATTTCATTATGCACACACACGTATTTTATCGAAGCAGCAATTACAATTTTCA

TGAGATTATATCAGTTTAATTTTAAATCTCAATTAGTTTC
```
